# Dynamic Changes in Meat Quality, Volatile Organic Compounds, and Microbial Community of Xiangxi Yellow Cattle Beef During Chilled Storage

**DOI:** 10.3390/foods14071139

**Published:** 2025-03-25

**Authors:** Liusha Kuang, Enqi He, Lei Zhou, Aihua Lou, Yan Liu, Wei Quan, Qingwu Shen

**Affiliations:** College of Food Science and Technology, Hunan Agricultural University, Changsha 410128, China

**Keywords:** beef, volatile flavor compounds, biogenic amines, microbial communities

## Abstract

Xiangxi Yellow Cattle, an indigenous Chinese livestock breed recognized for its exceptional nutrient composition and superior meat characteristics, has gained significant market preference among consumers. This investigation focused on evaluating physicochemical attributes, flavor development patterns, and bacterial population dynamics in chilled beef samples stored at 4 °C over an 8-day period. The research further examined interrelationships between meat parameters, predominant microbial species, and crucial aroma-active substances. Findings revealed a progressive elevation in lipid/protein oxidation levels, biogenic amine accumulation, and TVB-N values as microbial proliferation intensified toward the late storage phase. Microbial analysis demonstrated a final total viable count of 7.17 log10 (CFU/g), with bacterial community dominance distributed among *Firmicutes* (58.15%), *Proteobacteria* (29.56%), and *Bacteroidota* (12.05%). Notably, *Brochothrix thermosphacta* emerged as the primary spoilage organism in terminal storage phases. Volatile organic compounds (VOCs) in the beef during storage were analyzed, with a total of 66 compounds identified. The critical analysis identified 2,3-butanedione and 2-butanone as microbial metabolism-dependent substances showing strong correlations with meat quality parameters, emerging as freshness markers for beef evaluation. Importantly, the study highlighted the necessity for deeper investigation into microbial–VOC interactions, particularly considering the intricate bacterial ecosystems in aquatic environments. These outcomes enhance our understanding of spoilage mechanisms in Xiangxi Yellow Cattle beef while proposing practical approaches for microbial control in meat preservation systems.

## 1. Introduction

Beef serves as a nutrient-dense food containing premium-quality proteins, essential B vitamins, diverse minerals, vital trace elements, and important bioactive components. These nutritional elements collectively position beef as a valuable dietary option for fulfilling human physiological requirements [1]. Official statistics from the United States Department of Agriculture (USDA) indicate that worldwide beef consumption reached 57.485 million metric tons, with China representing nearly one-fifth of global demand. Nevertheless, beef products remain susceptible to quality deterioration through natural degradation processes, enzymatic activities, and bacterial proliferation occurring across supply chain stages from distribution to retail.

Microbial contamination is the most fundamental cause of beef spoilage, starting from the quarantine of raw materials, through the slaughtering, segmentation, acid discharge, packaging, transportation, storage, and sales processes. The proliferation of microorganisms throughout these phases may trigger biochemical alterations including protein structural breakdown, color alteration through pigment decomposition, and lipid oxidative damage, which collectively compromise product integrity and accelerate deterioration timelines in beef preservation. In addition, protein oxidation and lipid oxidation produce substances such as aldehydes, ketones, and alcohols, which affect the content of volatile flavor substances. Therefore, current research is focused on monitoring the quality changes of fresh meat after slaughter, during cold chain transportation, and throughout its shelf life. Wang Jue et al., recently conducted a study to determine the effect of different storage methods on the quality of chilled chicken meat. The results showed that the shelf life of chicken meat should not exceed 5 days when stored at 4 °C. This research highlights the importance of proper storage practices in order to maintain the quality and safety of meat products. Su Wang et al. investigated the effects of different cold chain logistics modes on the quality of fresh pork and found that suitable cold chain logistics modes can not only prolong the shelf life but also improve the quality of meat by reducing the growth of microorganisms [2].

Xiangxi Yellow Cattle have built a strong reputation in the consumer market for their rich nutrition, delicious meat, lack of strong or fishy odors, and popularity among local and national consumers. Presently, there are over 260,000 Xiangxi Yellow Cattle, producing 30,000 tons of beef in the central south region of China. However, similar to other meat products, Xiangxi Yellow Beef is also susceptible to quality deterioration caused by oxidation and microbial contamination, leading to economic losses. As a unique species in the Hunan province of China, the extent and impact of microbial contamination and quality changes post-slaughter are not yet well understood.

This investigation employed a multimodal analytical approach to monitor physicochemical transformations in refrigerated beef systems while establishing interconnections between biochemical alterations, aroma compound evolution, and microbial population dynamics. Through advanced molecular profiling techniques, we conducted comprehensive volatile organic compound analysis on Xiangxi Yellow Cattle hind cuts using HS-SPME-GC-MS integration (Agilent, Santa Clara, CA, USA), coupled with microbial community mapping through targeted sequencing of bacterial 16S rRNA (V3–V4) and fungal ITS genomic regions. Multivariate statistical modeling further elucidated tripartite relationships among microbial consortia, meat quality parameters, and sensory-relevant volatile markers. The integrated methodology reveals critical spoilage-associated microbiota while establishing predictive frameworks for freshness evaluation and optimized cold chain management of this premium beef product.

## 2. Materials and Methods

### 2.1. Chemicals and Reagents

Plate count agar (PCA) (Aoboxing Biotech Company in Beijing, China). Hydrochloric acid (HCl) solution for volumetric analysis (0.1 M; Anpel Laboratory Technologies, Shanghai, China). 5,5′-Dithiobis 2-nitrobenzoic acid (Aladdin Biochemical Technology Co., Ltd., Shanghai, China). 2,4-Dinitrophenylhydrazine (Abcam plc, Shanghai, China). All other reagents are made of analytically pure grades.

### 2.2. Sample Preparation

Experimental samples were sourced from the semitendinosus muscle of the premium hind cuts of Xiangxi Yellow Cattle supplied by Hunan Denong Animal Husbandry Co. (Huayuan, Changde, China), with slaughtering operations conducted under provincial food safety protocols. Following standardized processing, primal cuts underwent vacuum-sealed packaging and refrigerated transportation (4 °C) to research facilities. Initial preparation involved meticulous trimming of epimysial connective tissues, fascial membranes, and residual tendons to obtain standardized muscle specimens for analysis. Semitendinosus muscles were taken from the hind legs of 30 Xiangxi Yellow Cattle, and the muscle samples were cut into small pieces with an average weight of 200 g (10 cm × 10 cm × 3 cm) per piece, and the randomly drawn 30 muscles were divided into 5 groups. Each sample was sealed with plastic wrap and packaged in transparent high-barrier plastic bags (O_2_ permeability: 0.21 cm [3] (m^−2^ 24·h 0.1 MPa); water vapor permeability: 0.09 g (m^−2^ 24·h). The experimental meat specimens, encased in oxygen-permeable film, underwent controlled refrigeration at 4 °C until reaching spoilage endpoints. Systematic sampling was conducted at 48 h intervals over an eight-day observation period, with collected specimens chronologically coded as D0, D2, D4, D6, and D8 for analytical tracking. For each sampling time, three portions of bovine hind leg semitendinosus muscle were prepared. Each experiment was repeated three times.

### 2.3. Physicochemical Analysis

Physicochemical characterization involved dual analytical protocols: Sample acidity was determined with a calibrated portable pH meter (Testo 205, Shanghai, China), and surface chromaticity parameters (L*, a*, b*) were captured using a chroma analyzer (Konica Minolta CR-400, Tokyo, Japan). Triplicate measurements across three distinct surface points ensured data reliability. For exudate quantification, specimens underwent standardized conditioning at 4 °C for 24 h. following controlled equilibration, surface moisture was absorbed using sterile blotting media prior to gravimetric recording. Exudate losses were calculated through differential weight analysis between pre- and post-conditioning mass values, adhering to established meat science methodologies [4].

### 2.4. Total Viable Count (TVC) Analysis

The total viable count (TVC) was assessed using Tian’s method [5]. The microbiological evaluation was initiated with a standardized 1:10 dilution protocol, where 25 g test specimens underwent aseptic homogenization (225 mL 0.85% NaCl, 5 min vortex mixing). Quantitative microbial analysis employed PCA medium (Aoboxing Biotech, Beijing, China) through standardized pour-plate incubation (37 ± 1 °C, 48 h). Colony enumeration data underwent logarithmic conversion, expressed as Log CFU/g, with triplicate determinations per sample to ensure analytical precision.

### 2.5. Measurement of Lipid and Protein Oxidation

The levels of thiobarbituric acid reactive species (TBARS) in the hairtail muscle were measured by the oxidation products reacting with 2-thiobarbituric acid [6]. Oxidative damage biomarkers were quantified as MDA concentration in standardized units of mg per kg tissue mass, reflecting lipid peroxidation levels through malondialdehyde equivalence measurements.

Thiol group quantification in bovine myofibrillar proteins was conducted through DTNB colorimetric analysis following established protocols [7]. Analytical outcomes were standardized as nmol thiol equivalents per mg protein mass, providing precise measurement of oxidative modifications in muscle tissue.

The carbonyl content in beef protein was measured using 2,4-dinitrophenylhydrazine (DNPH), following the method outlined by Oliver, Ahn, Moerman, Goldstein, and Stadtman with slight modifications [8]. The content of carbonyls was expressed as nmol/mg protein.

### 2.6. Total Volatile Basic Nitrogen (TVB-N) Content Analysis

Volatile basic nitrogen quantification in bovine specimens was performed through a semi-micro nitrogen analysis protocol adapted from Zeng’s established methodology, ensuring precise measurement of protein degradation byproducts in chilled meat systems [9]. The beef was minced using a meat mincer (JYL-C022E, Joyoung, Jinan, China). A 40 g sample of minced beef was then transferred into a distillation tube, followed by the addition of 100 mL of distilled water, which was homogenized for 30 min. The prepared homogenate underwent alkalization through magnesium oxide supplementation (1 g) within a distillation apparatus. Subsequent TVB-N quantification was conducted via automated Kjeldahl analysis (Hanon K9840, Shanghai, China) following standardized protocols, with concentrations calculated as mg/100 g tissue mass serving as critical freshness indicators.

### 2.7. Biogenic Amine Analysis

According to the literature [10], biological amine extraction commenced with 0.5 g tissue homogenate undergoing acid hydrolysis in 20 mL 0.4 M HClO_4_ through sequential vortex mixing. The acid-soluble fraction was volumetrically standardized to 50 mL using matching molarity solvent, followed by refrigerated centrifugation (Eppendorf 5810R, Hamburg, German, 2263× *g*/10 min/4 °C). For derivatization, 1 mL clarified supernatant was supplemented with a 200 μL 2M NaOH, 300 μL NaHCO_3_ sat. solution, and 2 mL dansyl chloride (10 mg/mL), forming a reaction system incubated at 40 °C under light-protected conditions.

Following a 45 min equilibration phase at ambient conditions (25 °C), residual derivatization agents were neutralized through ammonia treatment (100 μL), with subsequent incubation under identical thermal parameters for 30 min. The reaction system underwent volumetric adjustment to 5 mL via acetonitrile dilution, followed by high-speed centrifugal clarification (11,180× *g*, 5 min) to isolate target analytes. The supernatant was filtered through a 0.22 μm filter membrane, and 20 μL of the filtrate was analyzed by high-performance liquid chromatography (1260; Agilent, Santa Clara, CA, USA) coupled with a TC-C18 chromatographic column (Agilent, 4.6 × 250 mm, 5 µm) at a flow rate of 0.9 mL/min using an isocratic elution with solvent A and solvent B. Solvent A consisted of acetonitrile, while solvent B was an acetonitrile–0.01M ammonium acetate solution (1:9/*v*:*v*) containing 0.1% acetic acid. Chromatographic detection parameters were optimized with UV monitoring configured at a 254 nm wavelength, while column thermal regulation was precisely maintained at 35 °C to ensure analytical reproducibility.

The Biogenic Amine Index (BAI) was calculated by summing the levels of putrescine, cadaverine, histamine, and tyramine according to the idea proposed by Triki et al. [11].

### 2.8. HS-SPME-GC-MS Analysis

The volatile compounds were extracted using headspace solid phase micro-extraction (HS-SPME) and were analyzed using a gas chromatography/mass spectrometry (GC/MS) system (QP-2010 Plus, Shimadzu Corporation, Kyoto, Japan), as described by Argyri et al., with some minor modifications. Homogenized beef aliquots (2 g) were transferred into 20 mL headspace vials with hermetic sealing. Volatile isolation employed a conditioned DVB/CAR/PDMS fiber (50/30 μm, Supelco, Saint Louis, MI, USA) under controlled thermal extraction (60 °C, 30 min). Post-extraction, the fiber underwent thermal desorption (250 °C, 5 min) in splitless injection mode for chromatographic separation and mass spectrometric detection.

The compounds were separated using a DB-5 column with dimensions of 30 m length, 0.25 mm inner diameter, and 0.25 μm film thickness. The carrier gas used was helium, with a flow rate of 1 mL/min. For the analysis of volatile compounds, the oven temperature was initially set at 40 °C for 3 min, followed by a 2 min maintenance period. The temperature was then programmed to increase at a rate of 4 °C/min until reaching 130 °C, after which it was further increased to 230 °C at a rate of 8 °C/min and held for 5 min. The interface temperature was maintained at 280 °C throughout the analysis. The mass spectrometer operated in electron ionization (EI) mode with an electron energy set at 70 eV and a scan range of 35–550 *m*/*z*. The temperatures of the mass spectrometer source and quadrupole were set at 230 °C and 150 °C, respectively. Compounds were compared against reference standards in the National Institute of Standards and Technology 20 database, with a positive and negative matching value exceeding 800 considered for qualitative analysis.

### 2.9. Microbial Diversity Analysis

To analyze microbial succession dynamics, refrigerated beef samples across distinct storage intervals underwent high-throughput sequencing. Genomic DNA extraction was performed with the AxyPrep DNA Gel Extraction Kit (Axygen Biosciences, CA, USA) per manufacturer protocols, followed by purity verification through 1.2% agarose gel electrophoresis. Target amplification focused on bacterial 16S rDNA hypervariable regions (V3-V4) using universal primers 338F/806R (sequences: 5′-ACTCCTACGGGAGGCAGCA-3′ and 5′-GGACTACHVGGGTWTCTAAT-3′). Purified amplicons were sequenced via Illumina platforms (MiSeq PE300/NovaSeq PE250) at Majorbio Bio-Pharm (Shanghai, China), with raw data processed through Fastp (v0.20.0) for demultiplexing and quality control.

### 2.10. Statistical Analysis

The results are presented as the mean ± standard deviation. Analysis of variance was conducted using SPSS software (v.26.0, IBM, Chicago, IL, USA), with significant differences defined as *p* < 0.05. The VOCs were compared against reference standards in the NIST 20 database. Following amplification, DNA fragments underwent high-throughput sequencing via Illumina platforms (MiSeq PE300/NovaSeq PE250, San Diego, CA, USA). Raw sequencing data were preprocessed through Fastp (v0.20.0) for demultiplexing and quality refinement. Interdisciplinary correlations between microbial taxa and flavor metabolites were visualized via heatmap analysis, employing Spearman’s rank correlation with dual thresholds: statistical significance (*p* < 0.05) and correlation strength (|r| ≥ 0.80). Color gradients represented correlation magnitudes, while asterisks denoted statistically significant associations.

## 3. Results and Discussion

### 3.1. Changes in Apparent Meat Quality

The pH value and drip loss are important indicators of freshness, ultimately affecting the final quality of the product [12,13]. As illustrated in Table 1, the pH values exhibited a consistent upward trend throughout the period of chill storage, peaking at D8 with a value of 6.89. The pH elevation observed in meat systems primarily stems from enzymatic deamidation processes in muscle proteins, occurring concurrently with microbial metabolic byproduct accumulation during storage [14]. Moreover, According to Liang et al. [15], the criteria for rating chilled fresh meat were divided into three categories: fresh meat (5.8–6.2), sub-fresh meat (6.3–6.6), and spoiled meat (6.7 or higher). By the eighth storage day, the experimental beef specimens attained a pH value of 6.87, entering the full spoilage phase—a deterioration pattern aligning with the microbial succession observations documented in Song’s seminal research on chilled meat preservation [16]. Furthermore, the drip loss of beef significantly increased to 6.197% at D8. Elevated pH levels in beef induce structural destabilization of proteins through the disruption of non-covalent interactions, notably affecting hydrogen bonding networks and hydrophobic associations. This results in changes to the secondary and tertiary structure of the protein, leading to denaturation and aggregation [17]. Consequently, this reduces the water-holding capacity of beef.

Meat color is a direct indicator of freshness and quality for consumers [18]. During chill storage, L* increased slightly for the first 4 days and then decreased significantly on Day 6 and Day 8. And b* values peaked on Day 2 and then gradually decreased, while the a* value increased significantly within the first 0–2 days of storage. This may be related to the oxidation process in beef, as changes in meat color are usually associated with the oxidation of myoglobin. The L* value stands for brightness, and a drop means that the surface of the beef is darkened, which may be due to oxidation causing myoglobin to convert to metryoglobin, resulting in a darker color. The a* value is considered the most important color parameter for fresh meat, as it reflects the level of myoglobin oxidation in the meat. In general, the higher the a* value, the fresher the meat [19].

### 3.2. Changes in Lipid and Protein Oxidation

**Figure 1:**
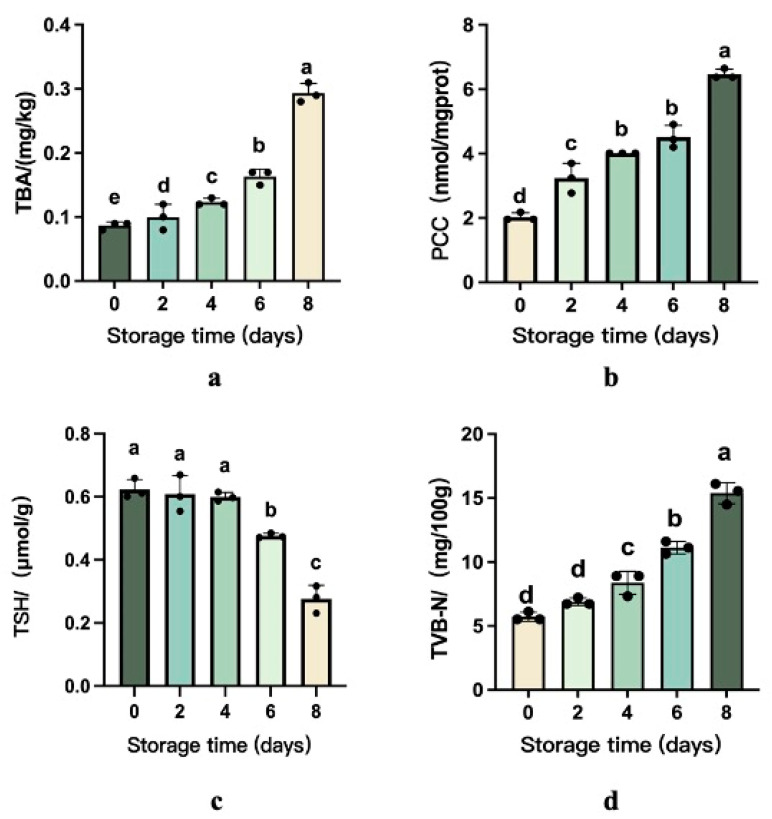
Changes in the content of TVB-N and level of protein and lipid oxidation of beef during chill storage (**a**–**d**). The different superscripts (a~e) in the columns denote differences (*p* < 0.05).

Throughout refrigerated storage, microbial proliferation drives a progressive quality decline in beef through dual oxidative pathways—lipid peroxidation and protein degradation. Thiobarbituric acid reactive substances (TBARSs) measurement emerges as the principal biochemical marker for quantifying fat oxidation severity, reflecting rancidity development in muscle tissues [20], which, in turn, can lead to a deterioration in the flavor and overall quality of the meat [21]. Based on Figure 1, the TBARS value of all samples significantly increased from 0.09 mg/kg to 0.292 mg/kg during chill storage.

As depicted in Figure 1, protein carbonyl levels in beef exhibited a progressive and statistically significant increase (*p* < 0.05) during refrigerated storage, ultimately reaching a 68.62% elevation relative to the baseline values observed at Day 0. The sulfhydryl groups in amino acids are easily attacked by reactive oxygen species radicals to form disulfide bonds, sulfonic acid, sulfurous acid, and other oxidation products [22]. The total sulfhydryl content in beef on the 6th to 8th days decreased significantly.

### 3.3. Total Volatile Basic Nitrogen (TVB-N) and Biogenic Amine Analysis

Total volatile basic nitrogen (TVB-N) quantifies protein degradation in meat products caused by enzymatic and microbial activity during storage, generating alkaline nitrogenous compounds like ammonia and biogenic amines. These volatile bases act as critical freshness biomarkers, with rising concentrations directly correlating to meat spoilage progression and reduced edibility [23].

TVB-N concentrations demonstrated a progressive elevation during chilled storage, escalating from 5.72 mg N/100 g (Day 0) to 6.89 (Day 2), 8.37 (Day 4), 11.13 (Day 6), and 15.38 mg N/100 g (Day 8), as illustrated in Figure 1. This sequential increment correlates directly with proteolytic degradation mechanisms driven by microbial proliferation and endogenous enzymatic activity [24]. In addition, Tahir HE et al. discovered a positive correlation between TVB-N and storage time. The TVB-N levels demonstrated progressive accumulation throughout storage, reflecting advancing spoilage [25]. Current Chinese food safety regulations (GB 2707-2016 [19]) establish 15 mg/100 g as the maximum permissible threshold for fresh/frozen beef. Experimental data revealed that samples stored through Day 8 exceeded this regulatory benchmark (15.38 mg/100 g), confirming terminal product deterioration.

Biogenic amines are a type of product obtained through the decomposition of proteins by tissue enzymes or microbial proteases [26]. Table 2 illustrates the changes in BA contents in beef during an 8-day storage period. Fresh beef inherently contains a certain amount of spermine (SPM) and spermidine (SPD) [27]. Prolonged refrigerated storage induced a significant accumulation of biogenic amines in beef samples, with putrescine (PUT) concentrations rising from 10.85 mg/kg to 169.27 mg/kg (*p* < 0.05), while cadaverine (CAD) progressed from non-detectable initial levels to 273.11 mg/kg by the study endpoint.

Tyramine (TYR) remained undetectable during initial refrigeration phases but exhibited exponential accumulation in terminal storage stages, culminating at 45.05 mg/kg. This delayed biogenic amine synthesis pattern aligns with empirical observations by Vinci et al. regarding microbial metabolite progression in chilled beef systems [28].

Meat with a Biogenic Amine Index (BAI) of over 50 mg/kg was classified as spoiled meat, according to studies by Triki et al. [11]. Based on this classification, beef stored at 4 °C exhibited signs of spoilage as early as Day 4, with the BAI index reaching 460.81 on Day 6.

### 3.4. Total Viable Count and Dynamic Analysis of Microbial Communities

During storage, microorganisms grow and multiply, producing toxic and harmful substances that can lead to beef spoilage [29]. Currently, the initial total viable count (TVC) of mesophiles and psychrophiles in beef was 2.81 log CFU/g on Day 0, but rapidly increased to 7.17 log CFU/g by Day 8. Microorganisms are present on the surface of beef during slaughter and transport, and during refrigeration, the loss of beef juices provides a moist and nutritious environment suitable for microbial growth. This rapid increase indicates that the beef was seriously contaminated during chilled storage, as TVC levels exceeding 7–8 log CFU/g are typically considered to indicate that the product has reached its sell-by date [30].

**Figure 2:**
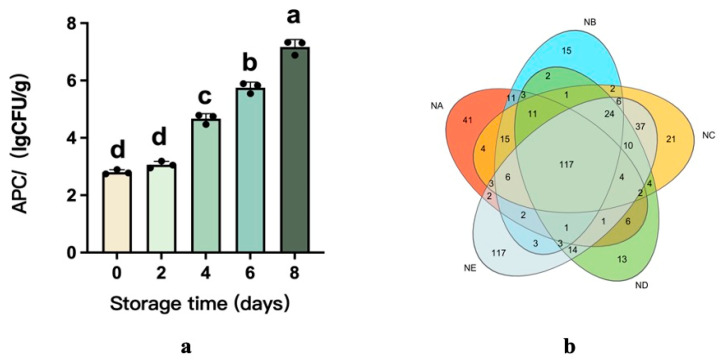
Evolution of total viable bacteria counts (TVCs) and the distribution of OTUs of the bacterial communities present in beef stored at 4 °C for 8 days (**a**,**b**). The different superscripts (a~d) in the columns denote differences (*p* < 0.05).

Furthermore, Illumina sequencing was used to characterize the bacterial diversity and community structure in the beef samples, which were classified into Group NA (0 d), Group NB (2 d), Group NC (4 d), Group ND (6 d), and Group NE (8 d). All sample groups achieved a Good’s coverage exceeding 99% (Table 3), demonstrating the dataset’s ability to represent bacterial communities accurately. Figure 2 illustrates that average Operational Taxonomic Unit (OTU) counts per group fluctuated between 155 and 198. A Venn diagram analysis identified 117 shared OTUs across all five groups, reflecting strong core community overlap. Unique OTU numbers showed distinct patterns: 41 on Day 0, 15 on Day 2, 21 on Day 4, 13 on Day 6, and 117 on Day 8. These results suggest dynamic community changes over time, with particularly high species richness observed on Day 8.

Alpha-diversity evaluates two key aspects of microbial communities: the total number of different species (species richness) and how evenly these species are distributed (species evenness) within a single sample [31]. Alpha-diversity is commonly quantified using metrics such as observed species counts and Chao, Shannon, ACE, and Simpson indices. In the case of the beef samples, the Simpson, ACE, and Chao indices ranged from 0.13 (D0) to 0.16 (D8), 195.65 (D0) to 243.37 (D8), and 191.57 (D0) to 241.47 (D8), respectively, indicating that community richness was higher on D8.

### 3.5. Changes in the Relative Abundance of Microbial Community Composition in Chilled Beef

**Figure 3:**
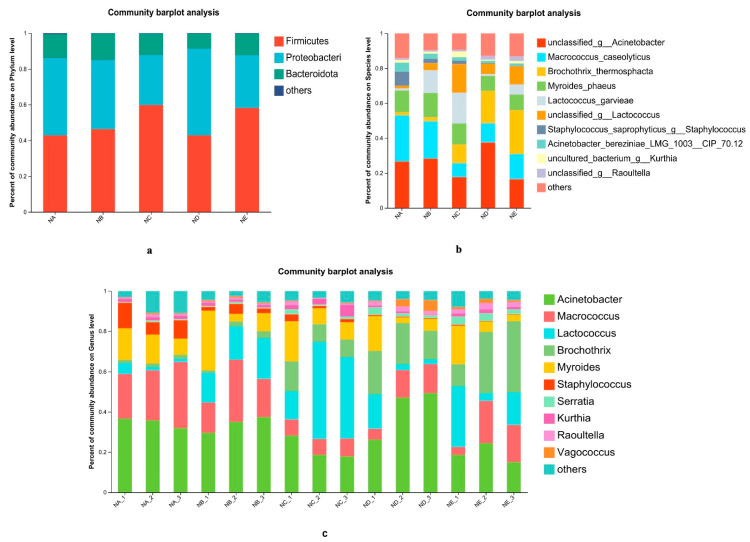
Microbial community composition of beef during chill storage. Relative abundance of bacteria in beef at the (**a**) phylum, (**b**) species, and (**c**) genus level.

Microbial community profiles in chilled beef were characterized over time at taxonomic levels ranging from phylum to species, as presented in Figure 3. This analysis revealed dynamic shifts in bacterial composition, with distinct changes observed in dominant taxa across different sampling intervals. Most components of the microbiota belonged to three major phyla (with relative abundances > 1%), including Firmicutes, Proteobacteria, and Bacteroidota. Firmicutes was the primary dominant bacterial phylum, with a relative abundance of 58.15%, followed by Proteobacteria and Bacteroidota with relative abundances of 29.56% and 12.05%, respectively, consistent with the results of [32]. In fungal communities at the genus level, the relative abundance of Brochothrix increased (*p* < 0.05), from 1.88% to 25.49%, respectively.

At the species level, the primary dominant bacterial species in beef samples was *unclassified g Acinetobacter,* with a relative abundance of 26.57%, followed by *Macrococcus caseolyticus* and *Myroides phaeus,* with 26.43% and 12.33%, respectively. Regarding the fungal communities at the species level, the relative abundance of *Brochothrix thermosphacta* increased (*p* < 0.05), from 1.86% to 25.19%, respectively. On the eighth day of storage, the primary dominant bacterial species in beef samples was *Brochothrix thermosphacta,* with a relative abundance of 25.19%, followed by *unclassified g Acinetobacter* and *Macrococcus caseolyticus,* with 16.49% and 14.32%, respectively. It has been recognized that *Brochothrix thermosphacta* is one of the dominant psychrophiles that cause spoilage in chilled meat, resulting in the production of volatile organic compounds with off-flavors, such as acetyl and diacetyl. This can cause spoiled meat to develop an unpleasant cheese or dairy flavor profile and, in most cases, result in green discoloration on the surface of chilled meat, with the production of green liquid [33]. *Macrococcus caseolyticus* will decompose the proteins in beef, releasing some compounds with special odors, such as amines, hydrogen sulfide, etc., which will lead to an unpleasant odor and reduce the organoleptic quality of the beef, making the beef soft and rotten, and causing it to lose its original elasticity and chewiness. There are also studies that show *Brochothrix thermosphacta* can produce a large amount of TVB-N. Notably, *Myroides phaeus* maintained a stable relative abundance throughout the storage period (Figure 3). This bacterium, classified under the *Myroides* genus, is recognized as a low-risk opportunistic pathogen typically found in environmental niches such as soil and water [34]. *Macrococcus* is an aerobic microorganism; in the early stage of cold storage, its abundance increased by utilizing the oxygen in the barrier film package. These findings suggest that *Brochothrix thermosphacta* growth persists even under refrigeration, potentially serving as a primary catalyst for accelerated beef spoilage during chilled storage.

### 3.6. Volatile Compound Analysis

**Figure 4:**
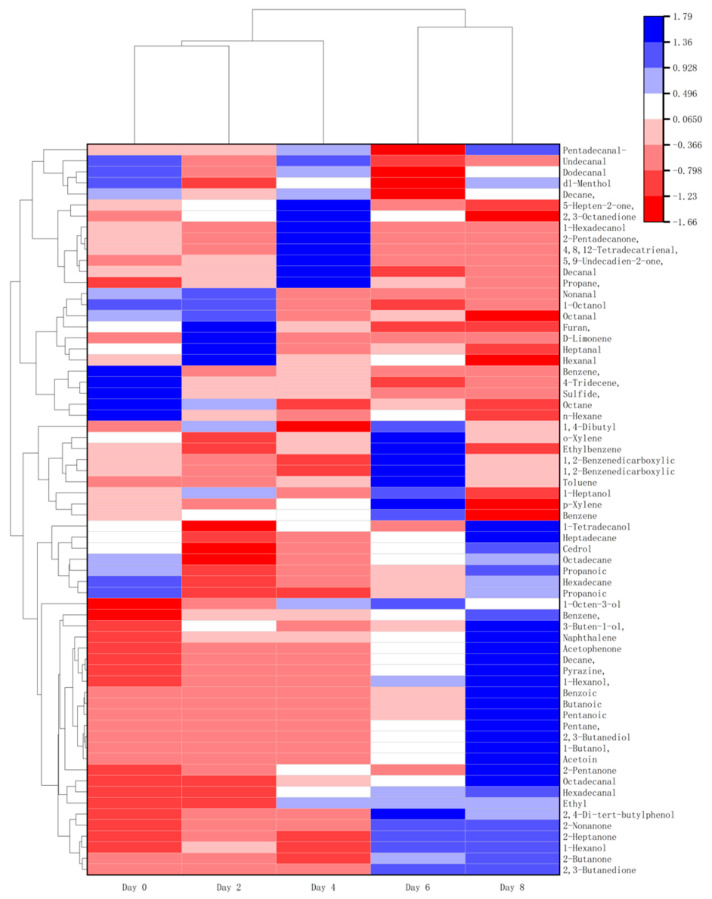
Heatmap showing the relative contents of various differential flavor compounds in beef during chill storage. Those with the highest abundant relationships are in red, while those with the lowest abundant relationships are in blue.

The process of microbial spoilage results in the sensory deterioration of beef during storage. Therefore, it is suggested that the growth of microbes is closely related to the sensory evaluation at the end of the shelf life. A total of 66 compounds were classified into ketones, alcohols, aldehydes, esters, hydrocarbons, and terpenoids based on their structural similarity, and were detected by HS-SPME-GC/MS (Table S1 and Figure 4). Alcohols, aldehydes, and esters are important sources of volatile flavor in meat products [35]. They are mainly generated from carbohydrate fermentation, reduction of methyl ketones, amino acid metabolism, lipid oxidation, oxidative cleavage of unsaturated fatty acids [36], and esterification [37].

To further clarify the differential flavor substances in the beef spoilage process, the Variable Importance in Projection (VIP) of PCA was utilized to screen the products [38], with the results displayed in Figure S2. A total of 10 different flavor substances were identified using VIP > 1 as the screening criterion. These include acetate, 2,3-butanedione, D-Limonene, 2-butanone, 1,2-Benzenedicarboxylic acid, bis(2-methylpropyl) ester, Nonanal, 2,3-butanediol, 5,9-Undecadien-2-one, 6,10-dimethyl-, (E)-, and Hexanal (Table 4). Aldehydes are one of the main products of fat oxidation in beef; the unsaturated double bonds in fatty acids will react with oxygen to form lipid peroxides, which are unstable and will be further decomposed to generate aldehydes and react with proteins and amino acids in beef. Alcohol compounds in the beef deterioration process are mainly related to microbial metabolism. Ketone compounds are mainly produced by the β-oxidation of fatty acids; ketone compounds have a special odor, and the presence of ketone compounds may provide a suitable environment for some microorganisms, promoting the further growth and reproduction of microorganisms. Among these, 2,3-butanedione, 2-butanone, and Hexanal have pungent and unpleasant odors, 2,3-butanedione has a rancid milk odor, 2-butanone has a fishy odor and an acetone-like odor, and ketones and aldehydes affect the flavor, oxidative stability, and color of beef. They play a crucial role in the formation of the foul smell of spoiled beef.

### 3.7. Correlation Analysis Between Dominant Microbiota, Meat Quality, and Key Volatile Compounds

**Figure 5:**
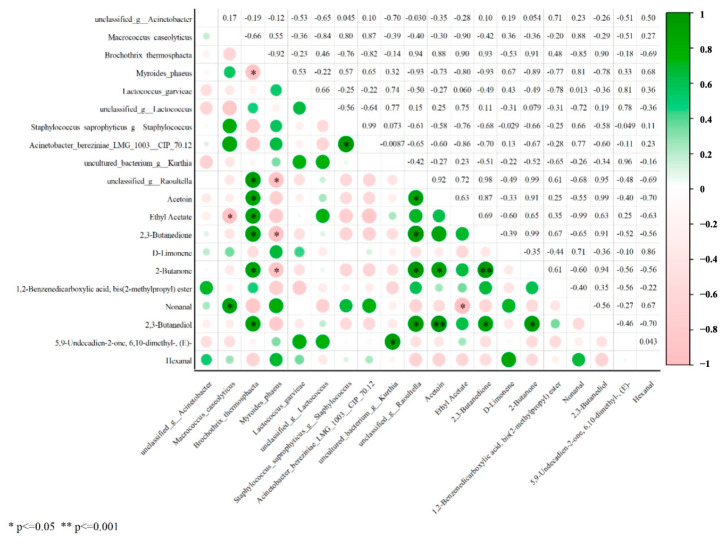
Correlation analysis between microbial genera and differential flavor compounds in beef during storage.

The undesirable odors generated during storage are related to bacterial metabolism [39]. In this study, the correlation between 10 key volatile compounds (VIP ≥ 1) and dominant microorganisms (the top 10 species levels in relative abundance) was analyzed. As shown in Figure 5, *Brochothrix thermosphacta* was significantly and positively correlated with Acetoin, Ethyl Acetate, 2,3-butanedione, 2-butanone, and 2,3-butanediol. Meanwhile, *Macrococcus caseolyticus* was significantly and positively correlated with Nonanal. It is worth noting that 2,3-butanediol and 2,3-butanedione have a pungent odor. Furthermore, the observed changes in flavor compounds and microorganisms at different storage times could provide valuable information for monitoring the quality of chilled beef.

**Figure 6:**
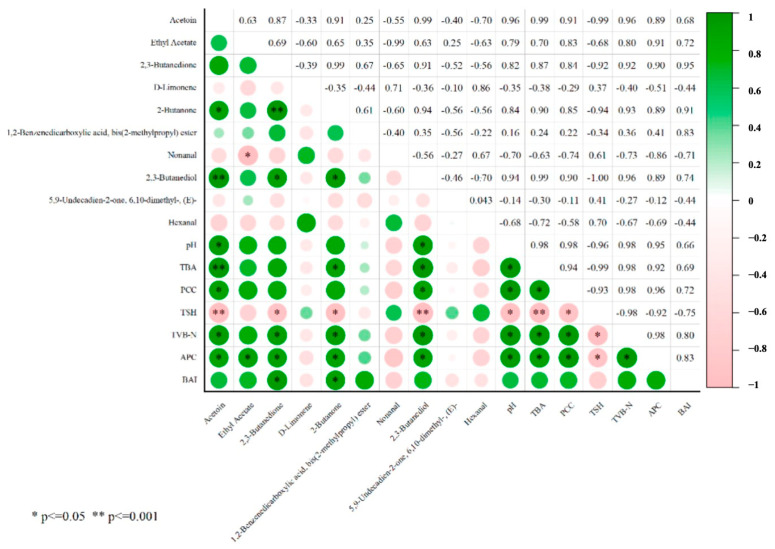
Correlation analysis between differential flavor compounds and meat quality in beef during storage.

Furthermore, the correlation between key volatile compounds and meat quality was analyzed (Figure 6). Acetoin, 2,3-butanedione, 2-butanone, and 2,3-butanediol were significantly correlated with changes in meat quality, such as pH values, lipid, and protein oxidation. In particular, 2,3-butanedione and 2-butanone exhibited a significant positive correlation with an increased TVB-N value and BAI index. This suggests that 2,3-butanedione and 2-butanone could be used as markers of quality changes and the growth of dominant spoilage microorganisms in chilled Xiangxi Yellow Cattle meat.

## 4. Conclusions

The present study provides information on the changes in the microbial community and physicochemical quality of Xiangxi Yellow Cattle meat preserved at 4 °C for 8 days. Chill storage had a significant effect on the overall meat quality, flavor composition, and microbial communities of the beef. Furthermore, the study analyzed the relationship between the dominant microbiota and key volatile compounds in aged beef through correlation analysis. It was found that 2,3-butanedione and 2-butanone were correlated with the growth and reproduction of *Brochothrix thermosphacta* and *Macrococcus caseolyticus* to some extent and were also associated with changes in meat quality. These findings provide valuable information for the quality control of chilled Xiangxi Yellow Cattle meat. Theoretical support was provided for the development of a low-cost and efficient early warning system for spoilage, and the identification of spoilage markers may provide a theoretical basis for subsequent methods of preserving beef and extending storage life. The use of natural antimicrobials and smart packaging technologies could significantly reduce meat losses (currently around 15% for frozen meat in China).

## Figures and Tables

**Table 1:** Changes in pH, surface color, and drip loss of beef during chill storage.

Storage Time	pH	Surface Color			Drip Loss (%)
		L*	a*	b*	
0	5.68 ± 0.06 ^c^	39.5 ± 3.77 ^a^	8.54 ± 0.49 ^b^	12.7 ± 1.39 ^ab^	0
2	5.88 ± 0.04 ^bc^	39.7 ± 5.69 ^a^	12.2 ± 2.50 ^a^	14.9 ± 0.51 ^a^	1.27 ± 0.41 ^b^
4	6.11 ± 0.28 ^b^	39.9 ± 0.65 ^a^	8.84 ± 0.86 ^b^	13.7 ± 0.78 ^ab^	2.60 ± 0.49 ^c^
6	6.18 ± 0.07 ^b^	36.2 ± 2.19 ^a^	6.46 ± 1.26 ^b^	11.5 ± 1.82 ^ab^	3.96 ± 0.22 ^d^
8	6.87 ± 0.26 ^a^	36.2 ± 2.57 ^a^	6.21 ± 0.45 ^b^	10.8 ± 1.73 ^b^	6.20 ± 0.60 ^e^

The different superscripts (a~e) in the columns denote differences (*p* < 0.05).

**Table 2:** Changes in the content of typical biogenic amines of beef during chill storage.

Day	PUT	HIS	SPD	SPM	TYR	CAD	BAI
0	10.85 ± 0.03 ^e^	6.38 ± 0.07 ^c^	13.7 ± 0.49 ^e^	106.9 ± 1.68 ^a^	-	-	17.23 ± 0.09 ^e^
2	14.35 ± 0.10 ^c^	6.43 ± 0.06 ^b^	13.8 ± 0.04 ^d^	102.9 ± 0.17 ^a^	-	-	20.78 ± 0.04 ^d^
4	11.67 ± 0.04 ^d^	6.24 ± 0.06 ^e^	14.20 ± 0.12 ^c^	89.4 ± 0.67 ^b^	-	43.26 ± 0.15 ^c^	61.19 ± 0.13 ^c^
6	169.3 ± 0.10 ^a^	8.78 ± 0.02 ^a^	38.3 ± 0.34 ^a^	60.5 ± 3.70 ^b^	9.64 ± 0.06 ^b^	273.1 ± 0.57 ^a^	460.81 ± 0.60 ^a^
8	92.8 ± 0.21 ^b^	6.25 ± 0.04 ^d^	15.49 ± 0.10 ^b^	85.7 ± 0.51 ^c^	45.1 ± 0.07 ^a^	192.9 ± 0.13 ^b^	337.10 ± 0.19 ^b^

The different superscripts (a~e) in the columns denote differences (*p* < 0.05).

**Table 3:** Phylotype coverage and α-diversity estimation of bacterial communities in hybrid grouper fillets during storage at 4 °C.

Sample	Shannon	Simpson	ACE	Chao	Coverage
NA (Day 0)	2.57	0.13	195.65	191.57	1.00
NB (Day 2)	2.53	0.14	189.81	184.41	1.00
NC (Day 4)	2.56	0.13	218.18	211.69	1.00
ND (Day 6)	2.34	0.18	197.60	189.61	1.00
NE (Day 8)	2.49	0.16	243.37	241.47	1.00

Shannon: the Shannon index of community diversity; Simpson: the Simpson index of community diversity; ACE: the ACE estimator; Chao: the Chao estimator; Coverage: the Good’s community coverage.

**Table 4:** Key volatile compounds of beef during chill storage.

Peak No.	Retention Time (min)	Average RI	CAS No.	Metabolite Name	Formula
1	3.653	714.81	513-86-0	Acetoin	C_4_H_8_O_2_
2	2.325	610.63	141-78-6	Ethyl Acetate	C_4_H_8_O_2_
3	2.149	596.89	431-03-8	2,3-Butanedione	C_4_H_6_O_2_
4	13.729	1027.47	5989-27-5	D-Limonene	C_10_H_16_
5	2.196	600.27	78-93-3	2-Butanone	C_4_H_8_O
6	37.095	1873.95	84-69-5	1,2-Benzenedicarboxylic acid, bis(2-methylpropyl) ester	C_16_H_22_O_4_
7	16.676	1104.49	124-19-6	Nonanal	C_9_H_18_O
8	5.573	799.54	513-85-9	2,3-Butanediol	C_4_H_10_O_2_
9	29.019	1457.42	3796-70-1	5,9-Undecadien-2-one, 6,10-dimethyl-, (E)-	C_13_H_22_O
10	5.593	802.21	66-25-1	Hexanal	C_6_H_12_O

## Data Availability

The original contributions presented in the study are included in the article, further inquiries can be directed to the corresponding author.

## Supplementary Materials

The following supporting information can be downloaded at https://www.mdpi.com/article/10.3390/foods14071139/s1, Table S1. Detailed information about volatile compounds from chilled beef by HS-SPME-GC-MS; Figure S1. Rarefaction curves (**a**) and Shannon curves (**b**) based on observed species; Figure S2. Loading plot (**a**), permutation test plot (**b**), and VIP score plot (**c**) for PCA analysis carried out on volatile profile data as obtained by the GC-MS in chilled beef.
