# Supplementary material for: Dynamic Changes in Meat Quality, Volatile Organic Compounds, and Microbial Community of Xiangxi Yellow Cattle Beef During Chilled Storage
Source: Foods. 2025 Mar 25;14(7):1139. doi: 10.3390/foods14071139 (PMC11988651; doi:10.3390/foods14071139)
Supplement: Supplementary file 1 [file foods-14-01139-s001.zip › foods-3526446-supplementary.pdf]

Table S1. Detail information about volatile compounds from chilled beef by HS-SPME-GC-MS.

Peak No.	Average Rt (min)	Average RI	CAS number	Metabolite name	Formula
1	2.149	596.89	431-03-8	2,3-Butanedione	C4H6O2
2	2.196	600.27	78-93-3	2-Butanone	C4H8O
3	2.199	598.93	110-54-3	n-Hexane	C6H14
4	2.325	610.63	141-78-6	Ethyl Acetate	C4H8O2
5	2.794	659.32	71-43-2	Benzene	C6H6
6	3.174	691.84	107-87-9	2-Pentanone	C5H10O
7	3.252	699.52	10152-76-8	Sulfide, allyl methyl	C4H8S
8	3.594	712.44	3877-15-4	Propane, 1-(methylthio)-	C4H10S
9	3.653	714.81	513-86-0	Acetoin	C4H8O2
10	3.965	731.48	763-32-6	3-Buten-1-ol, 3-methyl-	C5H10O
11	4.004	733.29	123-51-3	1-Butanol, 3-methyl-	C5H12O
12	4.074	735.68	564-02-3	Pentane, 2,2,3-trimethyl-	C8H18
13	4.679	763.02	108-88-3	Toluene	C7H8
14	5.524	800.27	111-65-9	Octane	C8H18
15	5.573	799.54	513-85-9	2,3-Butanediol	C4H10O2
16	5.593	802.21	66-25-1	Hexanal	C6H12O
17	7.12	846.26	2177-77-7	Pentanoic acid, 2-methyl-, methyl ester	C7H14O2
18	7.501	859.36	100-41-4	Ethylbenzene	C8H10
19	7.78	867.69	106-42-3	p-Xylene	C8H10
20	7.945	8.01	111-27-3	1-Hexanol	C6H14O
21	8.594	892.03	95-47-6	o-Xylene	C8H10

22	8.676	893.96	110-43-0	2-Heptanone	C7H14O
23	9.012	903.7	111-71-7	Heptanal	C7H14O
24	10.538	945.49	539-90-2	Butanoic acid, 2-methyl-, propyl ester	C8H16O2
25	11.493	11.71	111-70-6	1-Heptanol	C7H16O
26	11.976	981.67	3391-86-4	1-Octen-3-ol	C8H16O
27	12.163	986.58	585-25-1	2,3-Octanedione	C8H14O2
28	12.285	989.78	110-93-0	5-Hepten-2-one, 6-methyl-	C8H14O
29	12.37	992.01	3777-69-3	Furan, 2-pentyl-	C9H14O
30	12.381	992.3	95-63-6	Benzene, 1,2,4-trimethyl-	C9H12
31	12.832	1004.2	124-13-0	Octanal	C8H16O
32	13.729	1027.47	5989-27-5	D-Limonene	C10H16
33	13.867	1031.08	104-76-7	1-Hexanol, 2-ethyl-	C8H18O
34	13.94	1032.98	95-50-1	Benzene, 1,2-dichloro-	C6H4Cl2
35	14.883	1057.6	2847-72-5	Decane, 4-methyl-	C11H24
36	15.403	1066.66	98-86-2	Acetophenone	C8H8O
37	15.484	1073.24	111-87-5	1-Octanol	C8H18O
38	16.106	1089.64	1124-11-4	Pyrazine, tetramethyl-	C8H12N2
39	16.276	1095.67	821-55-6	2-Nonanone	C9H18O
40	16.676	1104.49	124-19-6	Nonanal	C9H18O
41	19.193	1172.74	1490-04-6	dl-Menthol	C10H20O
42	19.531	1181.68	91-20-3	Naphthalene	C10H8
43	20.393	1205.41	112-31-2	Decanal	C10H20O

44	20.705	1214.42	1002-11-5	Decane, 3-chloro-	C10H21 Cl
45	22.942	1277.51	41446-54-2	4-Tridecene, (Z)-	C13H26
46	23.826	23.92	112-44-7	Undecanal	C11H22 O
47	25.373	1348.61	109-15-9	Propanoic acid, 2-methyl-, octyl ester	C12H24 O2
48	26.108	1369.89	77-68-9	Propanoic acid, 2-methyl-, 3-hydroxy-2,2,4-trimethylpentyl ester	C12H24 O3
49	27.446	1406.64	112-54-9	Dodecanal	C12H24 O
50	29.019	1457.42	3796-70-1	5,9-Undecadien-2-one, 6,10-dimethyl-, (E)-	C13H22 O
51	30.69	1512.4	96-76-4	2,4-Di-tert-butylphenol	C14H22 O
52	32.617	1597.01	544-76-3	Hexadecane	C16H34
53	32.742	1603.11	77-53-2	Cedrol	C15H26 O
54	34.452	1697.24	629-78-7	Heptadecane	C17H36
55	34.097	1697.79	112-72-1	1-Tetradecanol	C14H30 O
56	34.694	1712.3	5444-75-7	Benzoic acid, 2-ethylhexyl ester	C15H22 O2
57	34.725	1714.32	2765-11-9	Pentadecanal-	C15H30 O
58	36.03	1798.08	593-45-3	Octadecane	C18H38

59	36.303	1817.33	629-80-1	Hexadecanal	C16H32O
60	36.668	1842.22	66408-55-7	4,8,12-Tetradecatrienal, 5,9,13-trimethyl-	C17H28O
61	36.713	1846.64	68607-88-5	2-Pentadecanone, 6,10,14-trimethyl-	C18H36O
62	37.095	1873.95	84-69-5	1,2-Benzenedicarboxylic acid, bis(2-methylpropyl) ester	C16H22O4
63	37.417	37.21	36653-82-4	1-Hexadecanol	C16H34O
64	37.741	1921.05	85-69-8	1,2-Benzenedicarboxylic acid, butyl 2-ethylhexyl ester	C20H30O4
65	38.366	1970.16	1962-75-0	1,4-Dibutyl benzene-1,4-dicarboxylate	C16H22O4
66	39.029	2022.96	638-66-4	Octadecanal	C18H36O

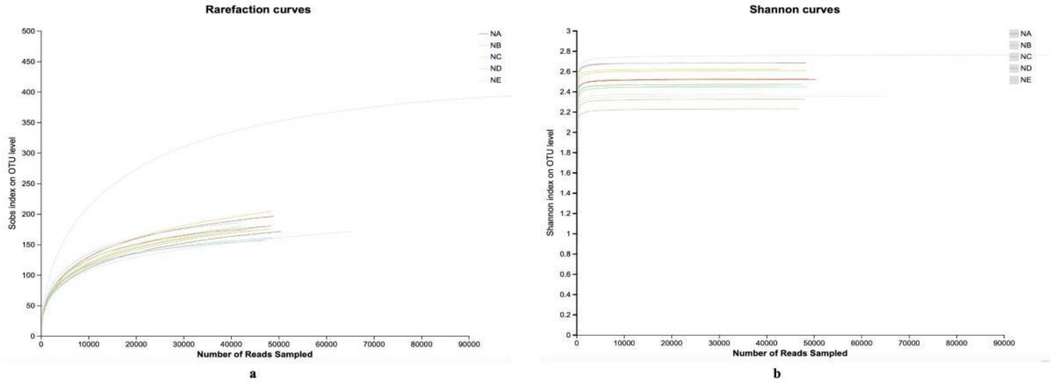

Figure S1. Rarefaction curves (a) and Shannon curves (b) based on observed species.

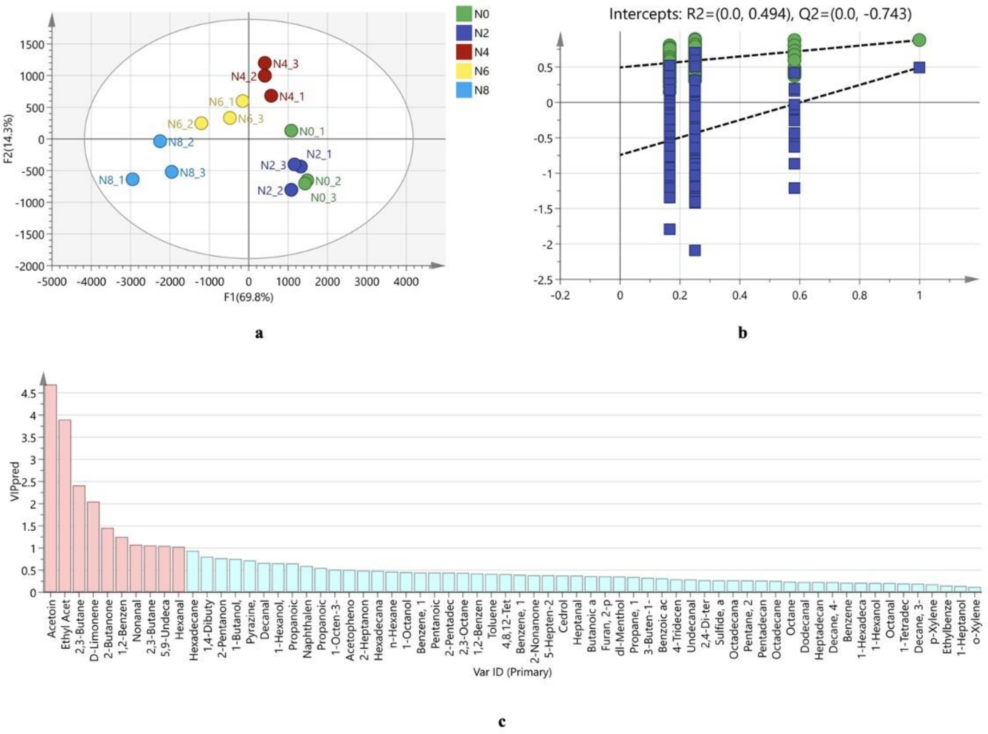

Figure S2. Loading plot (a), permutation test plot (b), and VIP score plot (c) for PCA analysis carried out on volatile profile data as obtained by the GC-MS in chilled beef.
